# Construction of a nomogram for predicting serum vitamin D deficiency in children/adolescents with new-onset type 1 diabetes: a single-center study in China

**DOI:** 10.3389/fped.2025.1554833

**Published:** 2025-05-30

**Authors:** Xin Yang, Hui Jiang, Min Liao, Meng Lin, Jin Wu

**Affiliations:** ^1^ Department of Pediatric Genetics, Metabolism and Endocrinology Nursing, West China Second University Hospital, Sichuan University, Chengdu, Sichuan, China; ^2^ Key Laboratory of Birth Defects and Related Diseases of Women and Children (Sichuan University), Ministry of Education, Chengdu, Sichuan, China

**Keywords:** vitamin D deficiency, children and adolescent, nomogram, predictive model, type 1 diabetes

## Abstract

**Objective:**

The vitamin D–type 1 diabetes (T1D) association has been debated in public health. The purpose of this study was to develop a vitamin D deficiency prediction model and investigate vitamin D deficiency risk factors in children and adolescents with new-onset T1D.

**Methods:**

A single-centre, retrospective analysis of paediatric patients (1–18 years) with new-onset T1D and initial 25-hydroxyvitamin D assessments was performed at a tertiary hospital in China between January 2020 and July 2024 (*n* = 353). The patients were divided into two groups according to whether their vitamin D deficiency exceeded 12 ng/ml. After identifying vitamin D deficiency risk factors in children/adolescents with new-onset T1D, a receiver operating characteristic (ROC) curve model was developed to predict the probability of vitamin D deficiency in these individuals. That model was represented with a nomogram. Calibration and clinical decision analysis curves were used to evaluate the model's effectiveness after internal validation via bootstrapping.

**Results:**

The prevalence rate of serum vitamin D deficiency among patients with new-onset T1D was 26.35% (93/353). Multivariate logistic regression analysis revealed that minority status (X1), weight (X2), diabetic ketoacidosis severity (X3), serum vitamin D testing season (X4), free triiodothyronine (X5), and high-density lipoprotein (X6) were closely associated with serum vitamin D deficiency development in children/adolescents with new-onset T1D (*P* < 0.05). The model was logit (P) =e^x^/(1 + e^x^), X = 4.626−1.878*X1−0.038*X2−0.821*X3−0.88*X4 + 0.351*X5 + 0.532*X6. The area under the curve (AUC) of the serum vitamin D deficiency predictive model among patients with new-onset T1D was 0.769 (95% CI = 0.711–0.826). The predicted probability's best cut-off value was 0.671.

**Conclusions:**

The established risk prediction model has good efficacy, providing a reference for screening high-risk vitamin D deficiency groups among children/adolescents with new-onset T1D and taking preventive and protective measures. The nomogram was developed based on a single-center cohort in China, and its generalizability needs further validation in more extensive populations.

## Highlights

• This study found that the prevalence of vitamin D deficiency [defined as 25-hydroxyvitamin D below 12 ng/ml (<30 nmol/L)] among children diagnosed with new-onset T1D was 26.35%.
• Vitamin D deficiency was more common among children and adolescents newly diagnosed with type 1 diabetes mellitus than in the general population.
• The established risk prediction model has good efficacy, providing a reference for screening high-risk vitamin D deficiency groups among children and adolescents with new-onset T1D.
• Lower 25-hydroxyvitamin D level was associated with minority, weight, diabetic ketoacidosis severity, season time of sampling, free triiodothyronine, and high-density lipoprotein.

## Introduction

Type 1 diabetes (T1D) is an endocrine disorder that occurs when pancreatic β cells cease generating insulin, usually as a result of autoimmune damage (1). Diabetes is one of the world's chronic illnesses endangering the health of children (2). In recent years, the incidence and prevalence of T1D have increased, from 0.51/100,000 people to 2.02/100,000 people, which places a considerable economic burden on families and society of children with T1D in China (3, 4). However, various recent studies have connected vitamin D status to diabetes aetiology, and research suggests that vitamin D deficiency can further, complicate the pathogenesis underlying diabetes mellitus and its consequences (5). There is considerable evidence that a deficiency of vitamin D, known colloquially as hypovitaminosis D, is a potential risk factor for T1D and is associated with adverse outcomes (6, 7). At present, the global prevalence of vitamin D deficiency is as high as 32.1% (8). Vitamin D deficiency has become a global public health issue worldwide since it has been attributed to cardiovascular, inflammatory, diabetes, and most autoimmune illnesses (9). Therefore, developing a model to predict vitamin D deficiency is crucial for identifying high-risk individuals among new-onset T1D patients.

Serum 25-hydroxyvitamin D [25(OH)D] issues have skyrocketed globally as a result of the increased interest in the potential pleiotropic effects of vitamin D (9). In this context, equipping specialists with clinical tools to distinguish between patients who are at high risk and those who are at very low risk of developing vitamin D deficiency may enable them to make better decisions about the individuals for whom it is necessary to measure the vitamin D concentration. Serum 25(OH)D concentrations are the most accurate way to determine the body's vitamin D status (10). Nevertheless, screening for serum 25(OH)D levels is not routinely included in usual T1D workups, and the importance of vitamin D deficiency among T1D patients is controversial (9).

Consequently, the purpose of this study was to develop a model for predicting the likelihood of vitamin D deficiency in children and adolescents with new-onset T1D and to lower the risk of developing vitamin D deficiency and identify high-risk individuals with it early.

## Materials and methods

### Patients and study design

This was a single-centre retrospective study. We retrospectively identified 353 consecutive paediatric patients newly diagnosed with T1D at a tertiary hospital (Sichuan, China, 104° latitude north) from January 2020 to July 2024, with measurements of serum 25(OH)D at the onset of diabetes. The inclusion criteria were as follows: (a) newly diagnosed with T1D according to expert consensus on the standardized diagnosis and management of T1D mellitus in Chinese children (2020): ① Symptoms of diabetes plus casual plasma glucose concentration ≥200 mg/dl (≥11.1 mmol/L); ② Fasting plasma glucose of ≥126 mg/dl (7.0 mmol/L) (fasting time 8–12 h); ③ OGTT 2 h value in venous plasma ≥200 mg/dl (≥11.1 mmol/L); ④ HbA1c ≥ 6.5% (11); (b) aged 1–18 years; and (c) complete case data with no missing values in key variables. The exclusion criteria the included the following: patients with type 2 diabetes mellitus, pharmaceuticals affecting vitamin D metabolism, and micro-or macrovascular problems.

### Collection and processing of samples

Newly diagnosed T1D patients were identified using an inpatient registration book. The following demographic and clinical data were collected through electronic medical records: demographic data, age, sex, minority, weight, height and first laboratory findings after admission. The laboratory findings included glycated haemoglobin (HbA1c), serum 25(OH)D, serum vitamin D testing season, pyruvic acid, total cholesterol, high-density lipoprotein (HDL) cholesterol, low-density lipoprotein (LDL) cholesterol, triglycerides, triglycerides, albumin, calcium, phosphorus, free Thyronine (T3), thyroxine (T4), free Triiodothyronine (FT3), free Thyroxine (FT4), thyroid stimulating hormone (TSH), diabetic ketoacidosis (DKA) severity, DKA was defined when the pH of the venous blood was <7.3 and ketonuria or ketonemia was confirmed when the serum glucose level was >200 mg/dl. DKA severity was classified as severe (pH < 7.1 or HCO3-<5 mmol/L), moderate (7.1 ≤ pH < 7.2 or 5 mmol/L ≤ HCO3-<10 mmol/L), or mild (7.2 ≤ pH < 7.3 or 10 mmol/L ≤ HCO3-<15 mmol/L) (11).

### Measurement of serum 25(OH)D

A chemiluminescent immunoassay was used to quantify the plasma levels of serum 25(OH)D, which included both 25(OH)D2 and 25(OH)D3. The cut-off for vitamin D deficiency and insufficiency may range depending on the indications of distinct organizations and communities; however, we applied the values recommended by the practical guidelines for clinical issues related to vitamin D nutrition in Chinese children, which defines vitamin D deficiency as 25(OH)D less than 12 ng/ml (<30 nmol/L), insufficiency between 12 and 20 ng/ml (30–50 nmol/L), sufficiency between 20 and 100 ng/ml (50–250 nmol/L), and toxicity greater than 100 ng/ml (>250 nmol/L) (12).

In addition, our patients were separated into two groups on the basis of their vitamin D levels. Individuals with 25(OH)D levels less than 12 ng/ml were classified as vitamin D deficient (group 1), whereas those with levels greater than 12 ng/ml were classified as nonvitamin D deficient (group 2). Considering that vitamin D levels are connected to sunlight exposure, which changes by season, the participants were separated into two groups on the basis of the 25(OH)D sample seasons: fall and winter (September 22–March 21) and spring and summer (March 22–September 21).

### Statistical analysis

The data were analysed using SPSS 17.0 software (version 17.0, SPSS Inc., Chicago, IL, USA) and DecisionLinnc software. Student's t test was used to compare normally distributed continuous data (means ± standard deviations). For categorical variables, data are expressed as numbers (percentages) and were compared by *χ*^2^ tests. Variables with of *P* < 0.05 in the univariate analysis were included in the logistic regression model for multivariate analysis to identify independent risk factors (13). The accuracy of vitamin D deficiency in children and adolescents with new-onset T1D was calculated using the receiver operating characteristic curve and the area under the curve. A higher AUC represents better discrimination ability of the prediction model. Calibration assesses a disease risk model's accuracy in forecasting the probability of an eventual event, as well as the consistency between the anticipated and actual risk levels. The model's calibration capabilities improved as the calibration curve became more similar to the standard curve. In addition, regression equations were calculated, and nomograms, ROC curves and calibration curves were plotted. A two-tailed *p* value less than 0.05 was considered to indicate statistical significance.

## Results

### Patient characteristics

The study population consisted of 353 patients (135 boys and 218 girls). The mean age of the participants was 8.49 ± 3.94 years (ranging from 1 to 18 years). Among the study participants, 93 had vitamin D deficiency (26.34%), 260 had no vitamin D deficiency (73.65%), 129 had vitamin D insufficiency (36.54%), and 131 had vitamin D sufficiency (37.11%). Patients were divided into two groups on the basis of their 25(OH)D levels [group 1: 25(OH)D <12 ng/ml; group 2: 25(OH)D ≥12 ng/ml]. The characteristics of the whole group and those of the two subgroups related to vitamin D status are shown in Table 1.

**Table 1:** Comparison of clinical characteristics between the two groups (*N* = 353).

Variable	Overall	Group 1 (*n* = 93)	Group 2 (*n* = 260)	Value	*P*
Age (y)	8.49 ± 3.94	9.75 ± 3.47	8.04 ± 4.01	3.651	0.000*
Gender (%)				1.915	0.166
Male	135 (38.24)	30 (32.25)	105 (40.38)		
Female	218 (61.76)	63 (67.74)	155 (59.62)		
Minority				9.615	0.002*
Han	331 (93.76)	81 (87.10)	250 (96.15)		
Other	11 (6.23)	12 (12.90)	10 (3.85)		
Weight (kg)	29.5 ± 15.51	33.41 ± 16.17	28.1 ± 15.05	2.766	0.006*
Height (m)	1.3 ± 0.24	1.38 ± 0.20	1.27 ± 0.24	3.683	0.000*
DKA severity					
No/mild	267 (75.64)	56 (60.22)	211 (81.15)	16.298	0.000*
Mode/severe	86 (23.36)	37 (39.78)	49 (18.85)		
Season				10.700	0.001*
Spring and summer	169 (47.88)	31 (33.33)	138 (53.08)		
Autumn and winter	184 (52.12)	62 (66.67)	122 (46.92)		
Initial HbA1c (%)	11.42 ± 2.82	12.22 ± 2.48	11.14 ± 2.88	3.232	0.001*
Triglycerides (mmol/L)	1.83 ± 2.94	2.06 ± 3.46	1.74 ± 2.73	0.898	0.370
Total cholesterol (mmol/L)	4.61 ± 1.57	4.88 ± 2.12	4.51 ± 1.31	1.951	0.052
HDL-cholesterol (mmol/L)	1.43 ± 0.50	1.28 ± 0.47	1.48 ± 0.63	−2.809	0.005*
LDL-cholesterol (mmol/L)	2.78 ± 1.071	2.94 ± 1.07	2.73 ± 1.07	1.684	0.094
Albumin (g/L)	42.57 ± 5.87	42.84 ± 8.54	42.47 ± 4.56	0.531	0.596
Pyranic acid (μmol/L)	62.08 ± 54.05	67.47 ± 55.71	60.16 ± 53.42	1.119	0.264
Calcium (mmol/L)	2.29 ± 0.28	2.27 ± 0.28	2.3 ± 0.28	−0.82	0.413
Phosphorus (mmol/L)	1.46 ± 0.41	1.34 ± 0.39	1.5 ± 0.41	−3.151	0.002*
T3 (nmol/L)	1.34 ± 0.56	1.16 ± 0.59	1.4 ± 0.54	−3.574	0.000*
T4 (nmol/L)	82.94 ± 30.27	78.37 ± 37.47	84.57 ± 27.14	−1.699	0.090
FT3 (pmol/L)	4.61 ± 1.51	4.05 ± 1.69	4.81 ± 1.39	−4.256	0.000*
FT4 (pmol/L)	14.66 ± 4.17	14.36 ± 5.21	14.77 ± 3.73	−0.829	0.408
TSH (mIU/L)	2.7 ± 2.76	3.03 ± 4.58	2.59 ± 1.70	1.319	0.188

Other: Yi, Tibetan and Buyi; DKA, diabetic ketoacidosis; HDL, high density lipoprotein; LDL, low density lipoprotein; T3, free thyronine; T4, thyroxine; FT3, free triiodothyronine; FT4, free thyroxine; TSH, thyroid stimulating hormone; DKA severity was classified as severe (pH < 7.1 or HCO3-<5 mmol/L), moderate (7.1 ≤ pH < 7.2 or 5 mmol/L ≤ HCO3-<10 mmol/L), or mild (7.2 ≤ pH < 7.3 or 10 mmol/L ≤ HCO3-<15 mmol/L); *: *p* < 0.05.

### Univariate analysis of vitamin D deficiency in children/adolescents with new-onset T1D

Univariate analysis revealed no significant differences in sex; triglyceride, total cholesterol, LDL, albumin, pyranic acid, calcium, T4, FT4, or TSH levels; or *P* values greater than 0.05 between the vitamin D deficiency group and the nonvitamin D deficiency group. However, there were significant differences in age, minority status, DKA severity on admission, serum vitamin D testing season, initial HbA1c, HDL, phosphorus, T3, and FT3, all with *P* < 0.05. See Table 1.

### Multivariate analysis of vitamin D deficiency in children/adolescents with new-onset T1D

The dependent variable was the prevalence of vitamin D deficiency, while the independent variable in logistic regression was the significance of univariate analysis. Age, minority status, DKA status at admission, serum vitamin D testing season, initial HbA1c, HDL, phosphorus, T3, and FT3 were found to be independent factors influencing vitamin D deficiency in children and adolescents with new-onset T1D. Multivariate logistic regression analysis was performed using the conditional forwards method. See Tables 2, 3.

**Table 2:** Assignments of categorical independent variables.

Variable	Assignment
Vitamin D deficiency	Group 1 (<12 ng/ml) = 0, Group 2 (≧12 ng/ml) = 1
Minority	Han = 1, Other Ethnicity = 2
Gender	Male = 1, Female = 2
Season	Spring and summer = 1, Autumn and winter = 2
DKA severity	No/mild = 1, Mode/severe = 2

**Table 3:** Results of binary logistic regression analyses of the potential risk factors for vitamin D deficiency in children/adolescents with new-onset T1D.

Parameter	β	SE	Wald c2	*P*	OR	95% CI	
Minority	−1.878	0.516	13.243	0.000	0.153	0.056	0.420
Weight	−0.038	0.009	17.410	0.000	0.963	0.946	0.980
DKA severity	−0.821	0.333	6.062	0.014	0.440	0.229	0.846
Season	−0.880	0.282	9.737	0.002	0.415	0.239	0.721
HDL	0.532	0.269	3.915	0.048	1.702	1.005	2.884
FT3	0.351	0.172	4.164	0.041	1.421	1.014	1.992
Constants	4.626	1.483	9.728	0.002	102.131	–	–

### Development and evaluation of a vitamin D deficiency prediction model

The following was the final prediction model of vitamin D deficiency in children and adolescents with new-onset T1D on the basis of the findings of logistic regression. logit (P) = e^x^/(1 + e^x^), X = 4.626−1.878*minority−0.038*weight−0.821*DKA severity−0.88*VD testing season + 0.532*HDL + 0.351*FT3.

### Multicollinearity test

For each independent risk factor in the regression model, the variance inflation factor (VIF) was calculated. There was no multiple collinearity among the variables, as indicated by the VIF values of weight (VIF = 4.702), minority (VIF = 1.049), DKA severity (VIF = 1.357), VD season (VIF = 1.047), HbA1c (VIF = 1.323), HDL (VIF = 1.069), phosphorus (VIF = 1.263), T3 (VIF = 3.723), and FT3 (VIF = 3.754). However, the VIFs for age (VIF = 9.914) and height (VIF = 10.773) were greater than 5 and were excluded.

### Evaluation and validation of the nomogram

The model that included all six variables was used to create a risk nomogram, which is a succinct tool for predicting the likelihood that children and adolescents with new-onset T1D will develop vitamin D deficiency.

**Figure 1:**
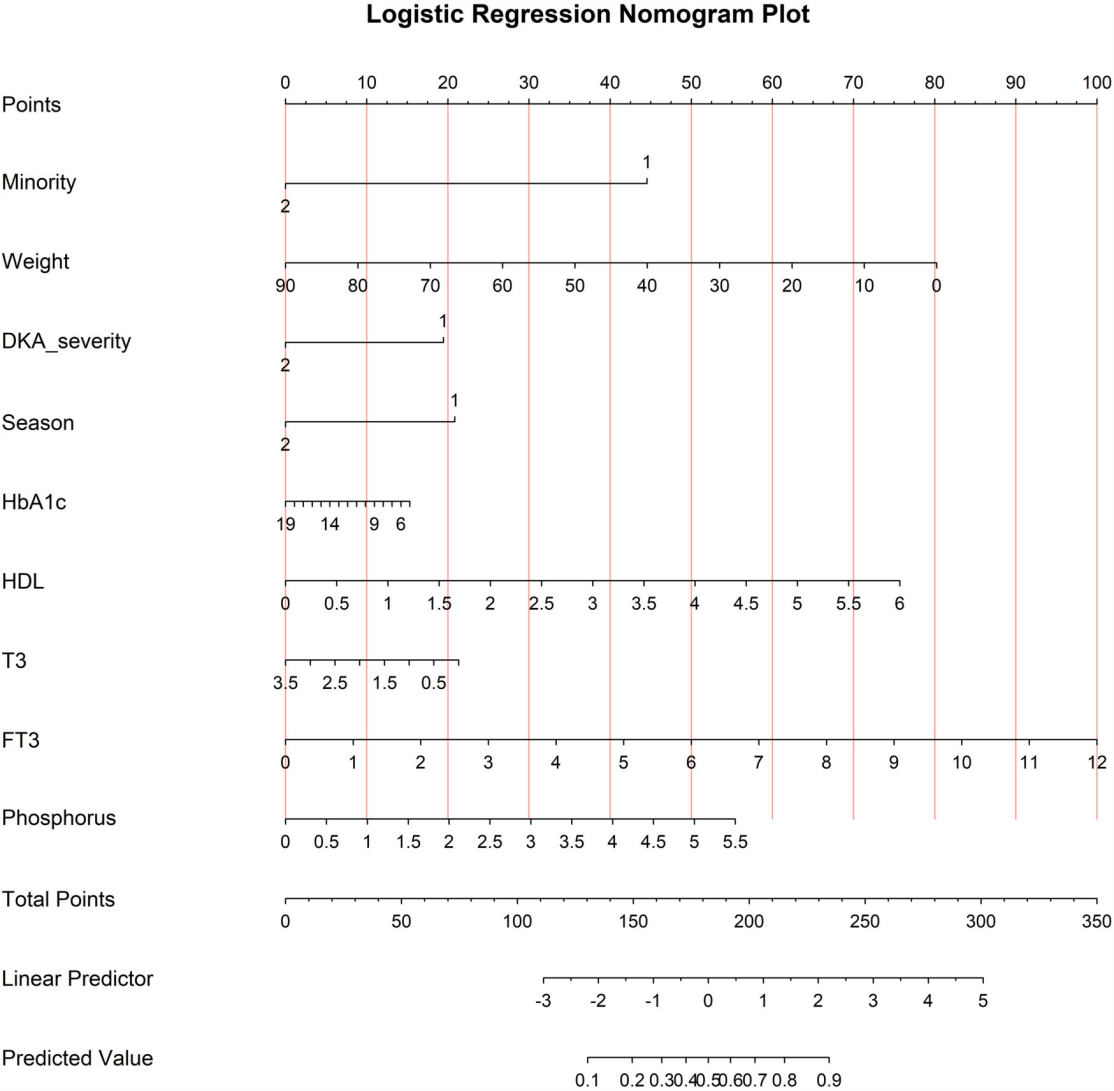
Prediction nomogram for vitamin D deficiency in children/adolescents with new-onset T1D.

The nomogram is divided into three sections: predicted probability, points, and predictive factors. Each factor's point reference line appears at the top, and the sum of all the factors' points appears at the penultimate line. Each factor's values are added to provide a total score that ranges from 0.2 to 0.9; larger scores suggest that children and adolescents with new-onset T1D are more likely to have vitamin D deficiency. A han Chinese child weighing 20 kg without diabetic ketoacidosis, during spring/summer seasons, with HDL levels of 3 mmol/L and FT3 levels of 5 pmol/L, the points assigned to each variable are 45, 62.5, 20, 20, 37.5, and 41.2 respectively. The total score of 226.2 corresponds to a position of 0.805 on the axis representing the risk of vitamin D deficiency, indicating an 80.5% risk of vitamin D deficiency in this child. See Figure 1.

**Figure 2:**
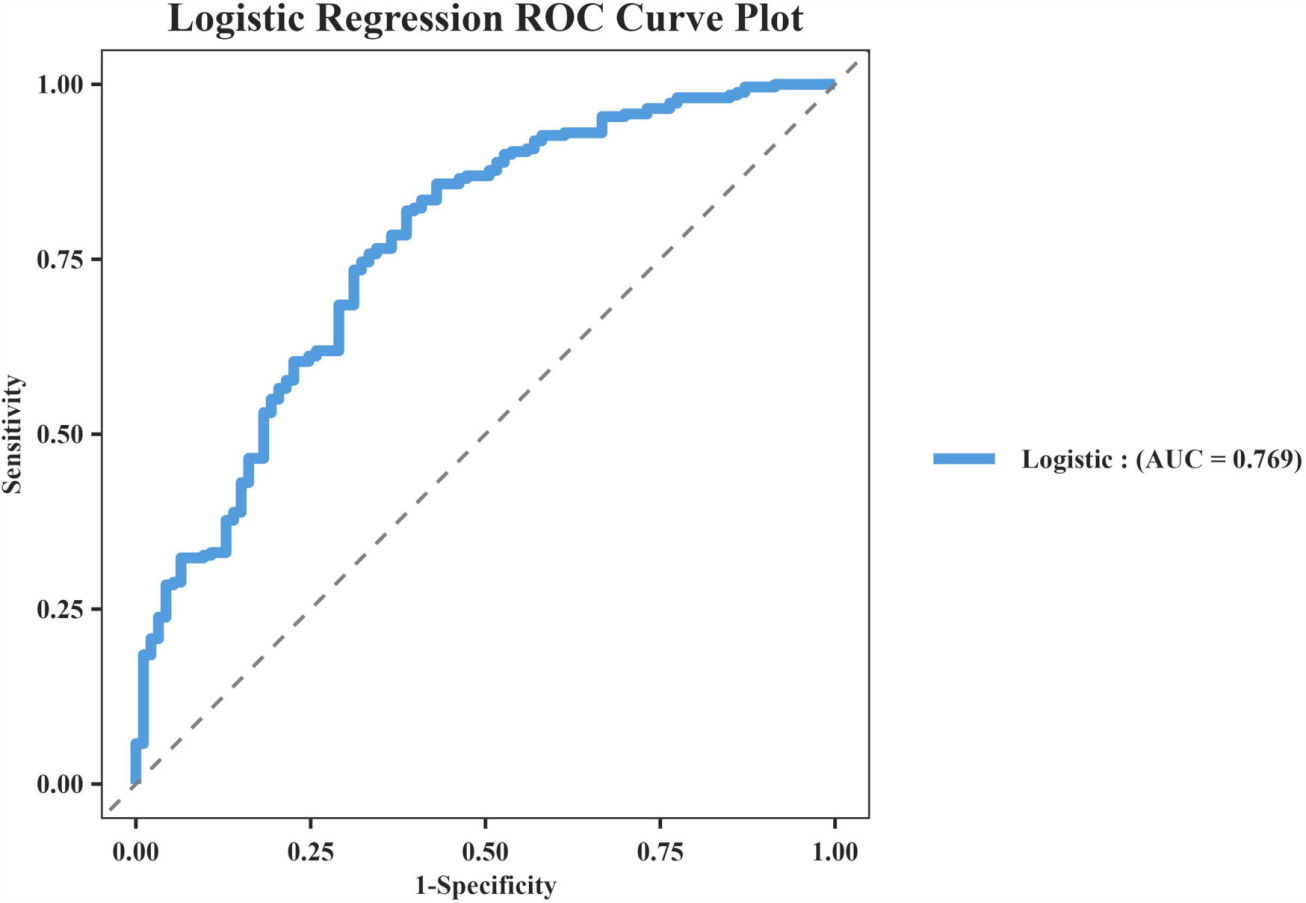
Receiver operating characteristic.

**Figure 3:**
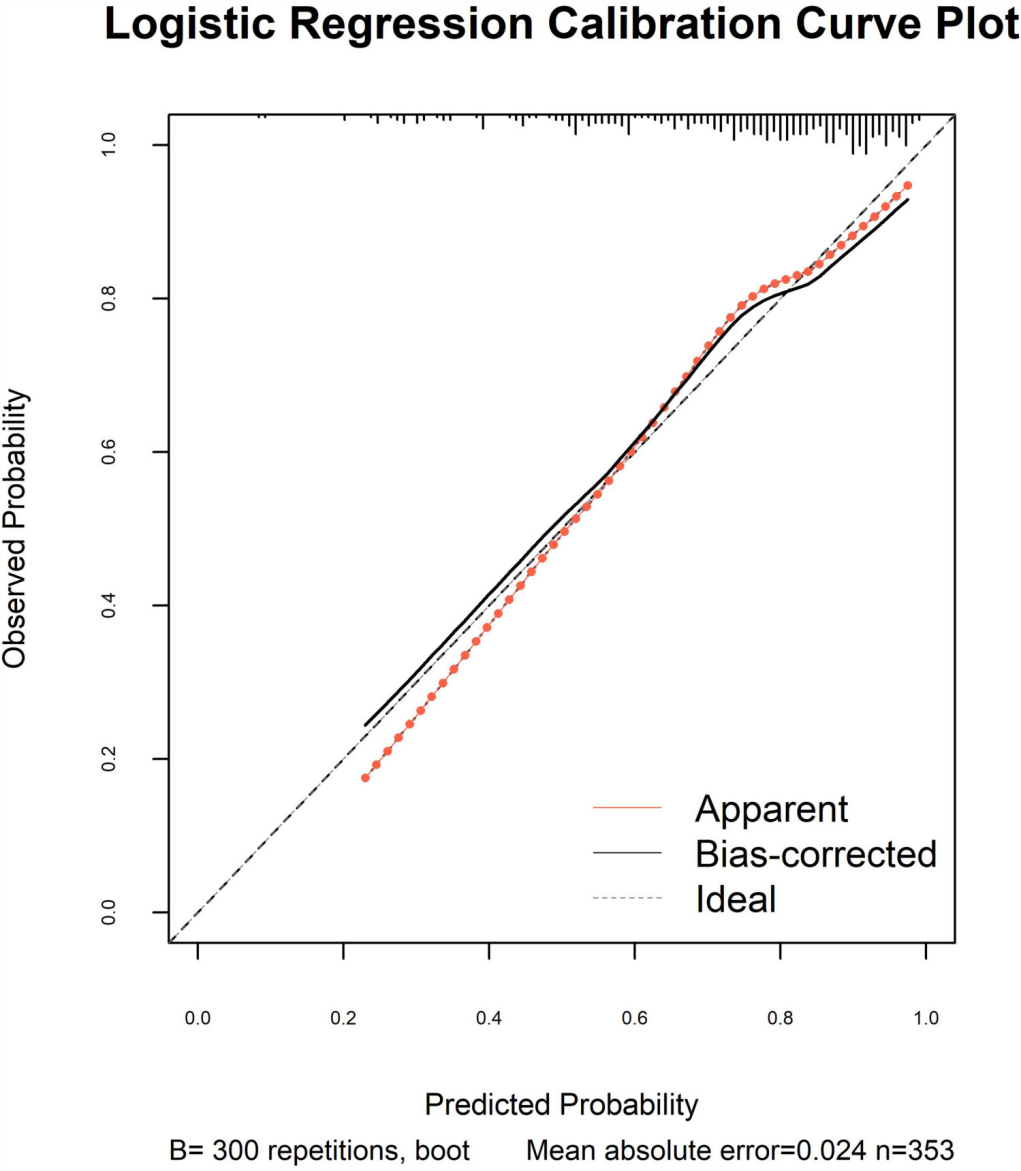
Calibration curve of nomogram.

**Figure 4:**
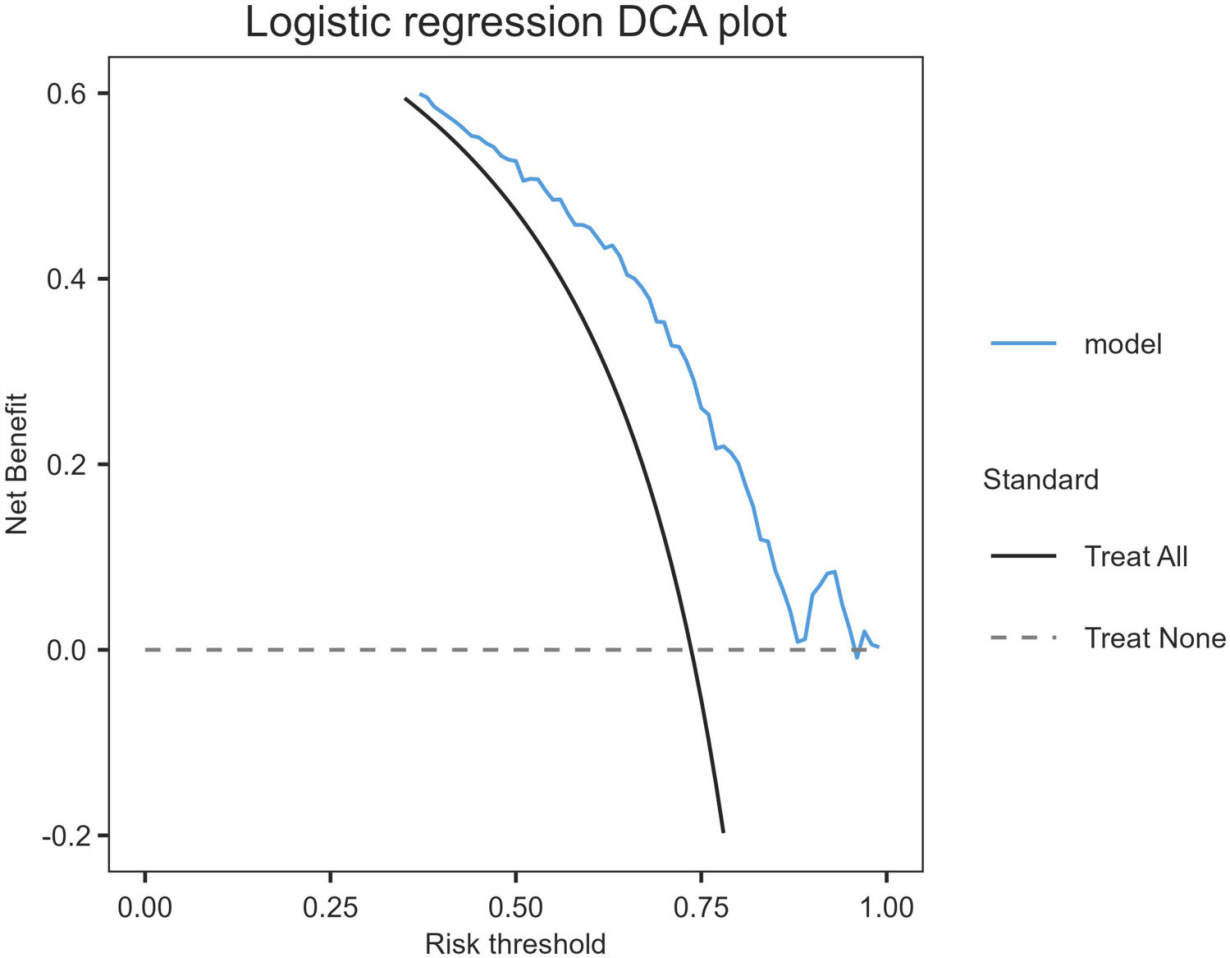
Decision curve analysis plot.

The model's discriminative capacity was assessed by computing the AUC and visualizing the ROC curve. The AUC for the predictive model of vitamin D deficiency was 0.769 (95% CI: 0.711–0.826; *P* < 0.05). The Youden index was 0.287 (sensitivity = 0.954 and specificity = 0.333), and the optimal cut-off value of the projected probability was 0.671. The aforementioned findings showed that the model can accurately identify vitamin D deficiency (Figure 2). The regression model fits well, as indicated by the nonsignificant Hosmer–Lemeshow test (*χ*^2^ = 8.792 and *P* = 0.36) (Figure 3). This indicates good goodness of fit. The calibration curves overlap with the 45 diagonal lines, indicating that the predictive model is accurate in its predictions. The utility and clinical application of the predictive model were assessed using DCA curves. DCA revealed that the model was highly useful up to a large threshold and was clinically meaningful. As illustrated in Figure 4, the model's high clinical usefulness in predicting serum vitamin D deficiency was proven by the substantial positive net benefit it showed regarding the risk of developing vitamin D deficiency.

## Discussion

The prevalence of vitamin D deficiency is generally considered high, even in the T1D population. According to the results reported above, 26.35% of our paediatric patients with new-onset T1D had a serum 25(OH)D level of less than 12 ng/ml. These findings are consistent with those of previous similar research (14). The current study clearly revealed that the prevalence of vitamin D deficiency was greater in new-onset T1D patients than in the general paediatric population (15). The interpretation of this phenomenon is that diabetes is considered to impair the hepatic synthesis of 25(OH)D and increase the catabolism of its active metabolite, hence exacerbating the problem of vitamin D deficiency (16). This finding also indicates that, given the growing knowledge of the importance of a lack of vitamin D in the aetiology of diabetes and its consequences. Therefore, it is necessary to construct a predictive model for vitamin D deficiency in T1D patients.

This study used stepwise logistic regression to develop a nomogram-based model to predict the risk of developing serum vitamin D deficiency in T1D patients. The feature variables included were age, minority status, weight, height, DKA severity status, serum vitamin D testing season, HbA1c, HDL-cholesterol, phosphorus, T3, and FT3. The ROC curve, calibration curve, and DCA were bootstrapped to demonstrate the model's good practicality, high consistency between actual values and forecasts, and good representativeness.

The univariate analysis identified 11 significant predictors out of 22 variables, yet only four predictors were ultimately included in the prediction model using multivariate analysis. All of these variables are simple to find in clinical settings, and together are capable of predicting vitamin D deficiency with good calibration and fair discrimination. This prediction model can assist in determining the risk of developing vitamin D deficiency in this high-risk population of individuals with newly diagnosed T1D.

Four predictors were identified in this model: minority status, vitamin D testing season, DKA severity status, and HDL—cholesterol. Minority and serum vitamin D testing season were specific predictors for vitamin D deficiency in children/adolescents with new-onset T1D. According to our findings, minority children and adolescents with new-onset T1D had lower serum 25(OH)D levels than patients with Han ethnicity did. There are several possible reasons for this finding, including a hypoxic environment (high-altitude geographical conditions with reduced oxygen availability), daily diet, clothes concealing participants’ ethnicity, etc (17) and the limitations of geographical location, as there were plateau-dwelling Sichuan individuals. Because of the high terrain and strong sunlight, plateau dwellers stay indoors for a long time to remain warm and avoid strong light, resulting in fewer outdoor activities (18). Additionally, the dates of the blood samples were recorded to evaluate the year-round variability in endogenous vitamin D production. Given that vitamin D levels are significantly higher in the summer and spring than in the winter and fall, our findings showed that the distribution of blood vitamin D status by season varies in T1D patients on the basis of the duration of sun exposure.

At present, the function of vitamin D in children and adolescents with T1D is still disputed (14, 19). In patients with new-onset T1D, the prevalence of medium/severe DKA-associated vitamin D deficiency was significantly higher in group 1 (39.78%) compared to group 2 (18.85%), indicating a potential association between low vitamin D levels and pre-DKA metabolic conditions. In the present study, we focused on new-onset T1D and various degrees of DKA, which has rarely been addressed (20). Given that vitamin D plays a part in glucose homeostasis, lower vitamin D levels are associated with more severe DKA and could indicate an aberration in glucose homeostasis (21). There are several possible reasons for this finding. First, it is well recognized that serum vitamin D protects against bacterial and viral infections, which may trigger diabetic ketoacidosis, and metabolic acidosis enhances urinary calcium excretion, reducing serum calcium and stimulating parathyroid hormone secretion, which expedites the conversion of 25 (OH) D3 to 1,25 (OH) 2D3 and depletes vitamin D reserves over time (22, 23). Second, animal studies have confirmed that chronic metabolic acidosis affects the activity of 1*α*-hydroxylase, resulting in the obstruction of the conversion of 25(OH)D3 to 1,25(OH)2D3 in the kidney. Moreover, in the state of ketoacidosis, vitamin D-binding protein may also be reduced, which would affect serum 25(OH)D level measurements (20).

HDL cholesterol was a specific predictor of vitamin D deficiency in newly diagnosed T1D patients. Vitamin D has anti-inflammatory effects, while low levels of HDL are often associated with chronic inflammatory states (24). Chronic inflammation may affect vitamin D metabolism and activity, resulting in vitamin D deficiency (25).

International research has established numerous predictive models for vitamin D deficiency based on various risk factors at an earlier stage, whereas the majority of domestic studies have been confined to investigating the risk factors associated with vitamin D deficiency, with no nomogram prediction models identified for vitamin D deficiency in children/adolescents with type 1 diabetes within China (8, 26, 27). This study develops a nomogram prediction model for vitamin D deficiency in newly diagnosed T1D patients, providing clinicians with a practical tool for the early identification of high-risk individuals. There were certain advantages to the nomogram that was suggested in this study. First, a nomogram for predicting the unresolved risk of developing vitamin D deficiency in children with new-onset T1D was established in the context of Chinese culture. Second, internal validation revealed that the nomogram had high calibration, discrimination, and clinical utility. Third, the majority of variables revealed by the nomogram are easily accessible and can reflect clinically important information, which can assist medical personnel in swiftly identifying major unresolved risk factors. Finally, given that this nomogram was developed on the basis of the Chinese population, it has good cultural representation.

This study has several limitations. Single-centre retrospective studies have some selection bias. Second, several confounding factors not included in the study, such as the food habits of the patients, may not have been sufficiently addressed. Third, information about the duration, dose, and compliance with previous vitamin D supplementation is unknown. Considering that the data were obtained from the patients’ medical records, it is impossible to rule out the possibility that some of the individuals had used vitamin D supplements in the past without records mentioning it. Furthermore, the prediction model might not be able to be used for nations with varying latitudes. For new study populations and environments, recalibrations can be necessary. The vitamin D deficiency prediction model must be externally validated in future research.

## Conclusion

Minority status, weight, DKA severity, season, HDL, and FT3 are independent risk factors for predicting vitamin D deficiency in new-onset T1D patients. The established risk prediction model has good efficacy, providing a reference for screening high-risk groups for vitamin D deficiency in children/adolescents with new-onset T1D. Paediatricians can use this information to calculate the probability of vitamin D deficiency and make timely interventions.

## Data Availability

The original contributions presented in the study are included in the article/Supplementary Material, further inquiries can be directed to the corresponding author.
